# Parents’ Judgments about the Desirability of Toys for Their Children: Associations with Gender Role Attitudes, Gender-typing of Toys, and Demographics

**DOI:** 10.1007/s11199-017-0882-4

**Published:** 2018-01-13

**Authors:** Marlene Kollmayer, Marie-Therese Schultes, Barbara Schober, Tanja Hodosi, Christiane Spiel

**Affiliations:** 1 Department of Applied Psychology: Work, Education and Economy, University of Vienna, Vienna, Austria; 2 Gillings School of Global Public Health, University of North Carolina at Chapel Hill, Chapel Hill, NC USA

**Keywords:** Parental attitudes, Toys, Gender socialization, Sex roles, Individual differences

## Abstract

Parental toy selection and responses to toy play are important factors in children’s gender socialization. Reinforcing play with same-gender-typed toys guides children’s activities and limits their action repertoires in accordance with gender stereotypes. A survey of 324 Austrian parents of three- to six-year-old children was conducted to investigate parents’ judgments about the desirability of different types of toys for their children and how these judgements relate to parents’ gender-typing of toys, gender role attitudes, and demographics (age, education, gender). Results show that parents rated same-gender-typed and gender-neutral toys as more desirable for their children than cross-gender-typed toys. The traditionalism of parents’ gender role attitudes was not associated with their desirability judgments of same-gender-typed toys, but was negatively related to their desirability judgments of cross-gender-typed toys. This indicates that egalitarian parents permit a greater range of interests and behaviors in their children than traditional parents do. Younger parents, parents with lower educational levels, and fathers reported more traditional gender role attitudes than did older parents, parents with higher educational levels, and mothers. However, no differences based on age, educational level or gender were found in parents’ judgments of toy desirability. The present study demonstrates that parents’ judgments about the desirability of toys for their children do not accurately reflect their gender role attitudes. This finding highlights the importance of simultaneously investigating different aspects of parents’ gender-related attitudes in order to gain a better understanding of parental transmission of gender stereotypes.

**Electronic supplementary material:**

The online version of this article (10.1007/s11199-017-0882-4) contains supplementary material, which is available to authorized users.

Toys play an important role in children’s gender socialization and educational pathways. They stimulate pretend play and social play as well as the development of cognitive skills (Cherney and Dempsey 2010). Many toys are stereotyped as appropriate for one gender rather than the other. Stereotypically feminine toys relate to nurturance, care, attractiveness, and beauty, whereas stereotypically masculine toys relate to technology, competition, aggression, construction, and action (Blakemore and Centers 2005; Campenni 1999). These features correspond to traditional gender stereotypes that are very stable over time (Ashmore and Boca 1979; Prentice and Carranza 2003). In general, men and women are thought to differ in terms of achievement-oriented traits, identified as agency, competence or instrumentality, and social- and service-oriented traits, identified as communion, warmth or expressivity (Kite et al. 2008). Men and boys are characterized as forceful, independent, and decisive (i.e., agentic attributes), whereas women and girls are characterized as kind, attractive, helpful, and concerned about others (i.e., communal attributes). These gender stereotypes lead to differential expectations of men and women, or boys and girls, regarding roles, interests, and skills and thus have a strong impact on educational and occupational careers (Fredricks and Eccles 2002; Kollmayer et al. 2016; Tiedemann 2000; Weisgram et al. 2011).

Gender-typed toy play leads to the promotion of different skills in boys and girls, with girls practicing communal roles and boys practicing agentic roles. This guides children’s activities in accordance with gender stereotypes and thus restricts their individual development potential (Cherney et al. 2003; Francis 2010; Jirout and Newcombe 2015; Li and Wong 2016). Whereas play with stereotypically masculine toys, such as blocks and toy soldiers, is generally associated with the development of spatial skills and more aggressive behavior (Hellendoorn and Harinck 1997; Jirout and Newcombe 2015), playing with stereotypically feminine toys, such as baby dolls and stuffed animals, is associated with more nurturing behavior (Caldera and Sciaraffa 1998; Liss 1983). Play with sexualized dolls, such as Fashion Barbies, is even associated with a narrowing of perceived career options in girls (Sherman and Zurbriggen 2014). In general, strongly gender-typed toys are less supportive of the development of children’s physical, cognitive, and artistic skills than are gender-neutral or moderately gender-typed toys (Blakemore and Centers 2005). Therefore, playing predominantly with same-gender-typed toys limits the development of children’s action repertoires in accordance with gender stereotypes and thus contributes to the perpetuation of gender stereotypes.

## Parental Influence on Children’s Toy Preferences

It is well known that children tend to prefer toys that are stereotypically identified with their own gender (Cherney and London 2006; Dinella et al. 2017; Fisher-Thompson 1993; Francis 2010; Ruble et al. 2006). This observed preference for same-gender-typed toys in children cannot be conceived in isolation from the role of parents in children’s gender socialization. Parents (i.e., legal guardians or closest caregivers, whether related by blood or not) are very important agents in children’s gender socialization, especially before children start attending school (Bussey and Bandura 1999; Maccoby 1998). Parental toy selection and responses to toy play are particularly important factors in children’s acquisition of gender stereotypes (Berenbaum et al. 2008; Kite et al. 2008; Wood et al. 2002), and preschoolers already apply gender stereotypes when asked with which toys their parents would want them to play (Raag and Rackliff 1998). Studies show that parents purchase more same-gender-typed toys than gender-neutral or cross-gender-typed toys for their children (Fisher-Thompson 1993; Kim 2002), although children seem to accept same-gender-typed, gender-neutral, and cross-gender-typed toys presented by their parents with equal joy (Idle et al. 1993). Thus, it is not surprising that parental toy choices are related to children’s play choices (Eisenberg et al. 1985). Still, the mechanisms by which parents’ gender-linked conceptions about toys are conveyed and translated to same-gender-typed toy preferences in children require further study. Moreover, the existing studies that examine parental toy choices are relatively dated which highlights the need for contemporary research on the topic.

According to social cognitive theory, gender development and differentiation is promoted by three major modes of influence, namely (a) modelling, (b) enactive experience, and (c) direct tuition (Bussey and Bandura 1999). With regard to toys, parents shape children’s preferences (a) by acting as models for symbolic play, (b) by rewarding desired toy choices and sanctioning undesired ones, and (c) by communicating gender-related knowledge and expectations about toys to their children. Parents who divide domestic labor in a traditional way model traditional gender roles that their children can rehearse in symbolic play with same-gender-typed toys. For example, girls can practice childcare by playing with dolls and boys can rehearse competitive behavior by playing with racing cars. Parental reactions to different toys also play an important role in shaping children’s toy preferences, and parents have been found to show more positive responses to toys gender-stereotyped for their child’s gender than to other toys (Caldera et al. 1989; Freeman 2007). Moreover, parents share their attitudes and expectations about gender and toys with their children through direct communication. Gelman et al. (2004) found that mothers who discussed a picture book depicting stereotypical gendered activities with their children affirmed gender stereotypes significantly more than they negated them. The three major modes of influence on gender development and differentiation, however, do not necessarily operate in concert (Bussey and Bandura 1999). On the one hand, parents are not always consistent in what they preach and practice. On the other hand, parents differ in terms of the gendered behavior they promote due to changing views on gender in certain quarters.

Parents’ attitudes about toys and gender play an important role in children’s gender socialization. Although research shows that parents’ behaviors do not always accurately reflect their attitudes (Halpern and Perry-Jenkins 2015; Perry-Jenkins and Crouter 1990), it is well known that parents’ gender-related attitudes are related to children’s gender-related cognitions (see meta-analyses by Degner and Dalege 2013 and Tenenbaum and Leaper 2002). Despite parents’ important role in shaping their children’s toy preferences and playing behavior, there is little research on the relationship between different aspects of parents’ attitudes and behaviors regarding toys and gender, namely their judgements about the desirability of different toys for their children, their gender-typing of toys, and their gender role attitudes (i.e. the degree to which they attribute traditional roles, interests, and abilities to men and women).

Parents with egalitarian gender role attitudes strive to avoid the transmission of gender stereotypes to their children by creating a non-stereotypical environment (Bem 1983), and in fact, egalitarian parents have been found to divide domestic labor more fairly and treat sons and daughters more similarly than traditional parents do (Crouter et al. 1995; Greenstein 1996; Updegraff et al. 1996). Blakemore and Hill (2008) found parents with more traditional gender role attitudes to be more likely to endorse same-gender-typed toys and less likely to endorse cross-gender-typed toys for their sons, whereas they were equally likely to endorse both types of toys for their daughters. Freeman (2007) found that preschoolers believe their parents are more supportive of their play with same-gender-typed toys than of their play with cross-gender-typed toys, regardless of parents’ gender role attitudes, which indicates mixed messages sent by parents. Thus, the relationship between parents’ gender role attitudes and their endorsement of different toys for their children remains to be clarified. In addition, no known research has addressed how parents’ gender-typing of toys relates to their gender role attitudes and judgments of toy desirability. Understanding the relations between different aspects of parents’ gender-related attitudes better is not only of theoretical importance but also a precondition for developing effective interventions.

## The Present Study

In the present survey study we aim to contribute to a better understanding of Austrian parents’ role in encouraging children’s gender-stereotyped toy play by examining different aspects of parents’ gender-related attitudes. These gender-related attitudes relate to the major modes of influence on children’s gender socialization proposed by social-cognitive theory (Bussey and Bandura 1999). First, we investigated parents’ judgments about the desirability of different types of toys for their children. Parents’ toy desirability judgments might be an indicator for their toy choices for their children as well as their reactions to children’s toy choices (i.e. enactive experience). Thus we hypothesized that parents would judge same-gender-typed toys as more desirable for their children than gender-neutral or cross-gender typed toys (Hypothesis 1). This hypothesis is consistent with previous findings that parents purchase more same-gender-typed than gender-neutral or cross-gender-typed toys for their children (Fisher-Thompson 1993; Kim 2002).

Second, we examined associations between parents’ judgments about the desirability of different types of toys for their children, their gender-typing of toys, and their gender role attitudes to find out if parents hold a constellation of consistent attitudes about gender. Parents’ gender-typing of toys might be an indicator for their gender-related communication about toys with their children (i.e. direct tuition), and parents’ gender role attitudes might relate to the gender roles they model (i.e. modelling). Therefore we expect more traditional gender role attitudes in parents to be associated with higher desirability judgments of same-gender-typed toys and lower desirability judgments of cross-gender-typed toys for their children (Hypothesis 2). Furthermore, we predict that more traditional gender role attitudes in parents will be associated with stronger gender-typing of toys (Hypothesis 3). To our knowledge, no research has examined the relationship between parents’ gender role attitudes and their tendency to see toys as gender-typed. Nevertheless, it seems plausible that parents who endorse traditional gender roles, compared to parents who reject them, will view toys promoting communal roles as more appropriate for girls and toys promoting agentic roles as more appropriate for boys.

Finally, we explored individual differences in parents’ gender-related attitudes in order to determine in which contexts children are particularly exposed to gender stereotypes. We investigated differences in parents’ judgments about the desirability of different types of toys for their children, gender-typing of toys, and gender role attitudes based on parents’ age, educational level, and gender, as well as their children’s gender. Specifically we hypothesized that younger parents, mothers, and parents with higher educational levels will report more egalitarian toy desirability judgments, less gender-typing of toys, and more egalitarian gender role attitudes than will older parents, fathers, and parents with lower educational levels, respectively (Hypothesis 4). Additionally, we expected that parents thinking of sons will judge cross-gender-typed toys as less desirable for their children than will parents thinking of daughters (Hypothesis 5).

## Method

### Participants

We recruited a total of 324 parents as they collected their child(ren) from Austrian preschools between February and May 2014. Parents participated in the study voluntarily and without receiving any incentive. Their mean age was 34.08 years (*SD* = 5.70, range = 19–52). The majority of the parents (256, 79%) were mothers, with a mean age of 33.61 years (*SD* = 5.34, range = 19–49). The surveyed fathers’ mean age was 35.85 years (*SD* = 6.66, range = 24–52). The parents were Caucasian, and most (*n* = 264, 81.5%) parents were born in Austria, 35 in Turkey, 12 in Germany, 3 in Romania, 2 each in Bosnia, Poland and Slovakia, and 1 each in Bulgaria, Macedonia, Hungary and Russia. They reported their highest educational attainment such that 33 (10.2%) had completed middle-school level education*,* 114 (35.2%) vocational training, 75 (23.1%) had a university entrance qualification, and 102 (31.5%) held a university degree. On average, the parents had 1.94 (*SD* = .85) children. We ensured that only one parent per child took part in the study. Parents were asked to think of one of their own preschool children aged three to six years old when completing the questionnaire. Approximately half of the parents (155, 47.8%; where 35, 22.6%, were fathers) thought of a son (*M*_age_ = 5.06 years, *SD* = 1.14), whereas 169 (52.2%; where 33, 19.5%, were fathers) of the parents thought of a daughter (*M*_age_ = 4.83 years, *SD* = 1.16) when answering the questionnaire.

### Materials and Procedure

#### Pilot Study

Before conducting the main study, we created an up-to-date set of gender-typed and gender-neutral toys in a pilot study. The pilot study included 29 community participants (62.1% female) with a mean age of 33.79 years (*SD* = 12.39*,* range *=* 19–58), 10 (34.5%) of whom were parents. The participants rated 42 toys that had been utilized in four former studies on the gender-typing of toys (Blakemore and Centers 2005; Campenni 1999; Kanka et al. 2011; Wagner et al. 2009) as to whether they were appropriate for boys, girls, or both on a visual analogue scale (0 = for girls, 50 = for both, 100 = for boys). The five toys most strongly associated with girls, the five toys most strongly associated with boys, and the five toys least associated with either girls or boys (i.e. with ratings closest to 50) were selected for the main study. The five toys most strongly associated with girls (*M* = 18.25, *SD* = 11.92) were a Barbie doll, wooden beads, doll clothes, a doll house, and a doll buggy. The five toys most strongly associated with boys (*M* = 75.92, *SD* = 12.56) were a Transformer, a truck, a helicopter, boxing gloves, and a Matchbox car. The five toys least associated with either boys or girls (*M* = 50.06, *SD* = 2.00) were a magnetic drawing board, an alphabet puzzle, plasticine (a type of modeling clay), a xylophone, and a doctor’s kit. The specific ratings for each toy can be found in an online supplement (see Table 1s). We conducted three one-sample *t*-tests against the mean of 50 to verify that the three sets of toys are statistically consistent with their labels as stereotypically feminine, *t*(28) = 14.34, *p* < .001, *d* = 2.66, masculine, *t*(28) = 11.12, *p* < .001, *d* = 2.06, and neutral, *t*(28) = .17, *p* = .868, *d* = .03. The 15 toys selected for the main study were digitally photographed and edited using an image editing program (GIMP 2.8.10). To avoid intervening effects of gender-typed colors (see Weisgram et al. 2014), we replaced the colors pink and blue with less gender-typed colors, mainly yellow and green.

#### Main Study

Participants either answered a paper-and-pencil questionnaire on the spot, sent it back to us after completing it at home, or completed an online version of the questionnaire. In the first part of the questionnaire, participants were asked for sociodemographic information about themselves (gender, age, educational attainment) and their child(ren) (number of children; age and gender of each child). Participants were asked to think of their three- to six-year-old child when answering the questionnaire. Parents with more than one child within this age range were asked to think of the oldest of these children and to indicate this child’s birthday and gender. In the second part of the questionnaire, the 15 toys selected from the pilot study were presented to the participants with photos and names. Participants were asked to familiarize themselves with the toys because they would encounter them repeatedly while answering the questionnaire.

#### Toy Desirability Judgments

In the third part of the questionnaire, we assessed parents’ judgments about the desirability of the toys for their children by asking them to indicate how desirable they found each of the 15 toys for their child. Parents rated the toys on a 7-point Likert scale ranging from 1 (*not desirable at all*) to 7 (*very desirable*). Three scales were formed by grouping together the toys of each type and averaging the ratings for the five toys within each grouping wherein higher scores indicated greater desirability. The first scale represented parents’ desirability judgments about stereotypically feminine toys, the second scale represented their desirability judgments about stereotypically masculine toys, and the third scale represented their desirability judgments about gender-neutral toys. Internal consistencies of the scales were good for the gender-typed toys (α_feminine toys_ = .83; α_masculine toys_ = .87) and satisfactory for the gender-neutral toys (α = .73). Linking the first two scales with the child’s gender resulted in one scale depicting parents’ desirability judgments about same-gender-typed toys (i.e. stereotypically feminine toys for girls and stereotypically masculine toys for boys) and another scale depicting their desirability judgments about cross-gender-typed toys (i.e. stereotypically masculine toys for girls and stereotypically feminine toys for boys) with reference to their own child.

#### Gender-Typing of Toys

In the next part of the questionnaire, we assessed the degree to which parents viewed toys as gender-typed. Participants were asked to rate the 15 toys as to whether they were appropriate for boys, girls, or both on a visual analogue scale (0 = *for girls*, 50 = *for both*, 100 = *for boys*). We again conducted three one-sample *t*-tests against the mean of 50 to verify that the three sets of toys are statistically consistent with their labels as stereotypically feminine, masculine, and neutral. The mean ratings of the stereotypically feminine toys (*M* = 29.05, *SD* = 14.21), *t*(324) = 26.53, *p* < .001, *d* = 1.47, and the stereotypically masculine toys (*M* = 71.81, *SD* = 15.88), *t*(324) = 24.73, *p* < .001, *d* = 1.38, were both significantly different from 50. Due to the large sample size and the low standard deviation, the mean rating of the gender-neutral toys (*M* = 49.50, *SD* = 2.49) was also significantly different from 50, *t*(324) = 3.63, *p* < .001, *d* = .20, in the direction being slightly more feminine. However, the small effect size indicates that this difference, although statistically significant, is not very meaningful and is unlikely to be detected in everyday life (Cohen 1977). The specific ratings for each of the 15 toys can be found in an online supplement (see Table 2s).

Two scales resulted, one capturing parents’ gender-typing of stereotypically feminine toys (α = .76) and the other assessing parents’ gender-typing of stereotypically masculine toys (α = .81). For better comparability in further analyses, the values for stereotypically feminine toys were subtracted from 100. This conversion resulted in higher values indicating stronger gender-typing of toys for both stereotypical girls’ toys and stereotypical boys’ toys. These values were combined to form a gender-typing of toys scale (α = .86), which measures parents’ overall tendency to see stereotypically feminine toys as appropriate for girls and stereotypically masculine toys as appropriate for boys.

#### Normative Gender Role Attitudes (NGRO)

The last part of the questionnaire assessed parents’ gender role attitudes. Participants completed the Questionnaire on Normative Gender Role Attitudes (NGRO; Athenstaedt 2000). The questionnaire consists of 29 items formulated as descriptive and prescriptive statements dealing predominantly with men’s and women’s suitability for different roles and occupations as well as their domestic and family responsibilities. Sample items include: “Every boy should own a doll” (reversed scored), “Girls like helping with the household more than boys,” and “Male police officers provide a stronger sense of security than female police officers.” The answering format used a 7-point Likert scale ranging from 1 (*strongly disagree*) to 7 (*strongly agree*). The NGRO scale allows respondents to be placed on a continuum from traditional to egalitarian gender-role attitudes. Higher averaged values indicate a more traditional view of gender roles. The internal consistency of the scale was strong (α = .89).

## Results

### Preliminary Analyses

We first conducted preliminary analyses to find out whether it would be legitimate to use the newly formed scales for parents’ desirability judgments about same- and cross-gender-typed toys and their overall gender-typing of toys in further analyses. We examined associations between the original scales (namely parents’ judgments about the desirability of different types of toys for their children, their gender-typing of stereotypical girls’ and boys’ toys, and their NGRO) separately for parents thinking of sons and parents thinking of daughters (see Table 1 for correlation coefficients). We expected correlations with scales in which parents judged the desirability of gender-typed toys for their children to be in opposite directions for sons and daughters. Apart from that, we did not expect different relationship patterns for parents of daughters and parents of sons. Moreover, we expected parents’ gender-typing of stereotypically feminine and stereotypically masculine toys to be highly correlated. Otherwise, the use of the newly formed scales would not be legitimate. Apart from the expected differences, we found almost identical relationship patterns for parents of sons and parents of daughters, albeit slightly higher correlation coefficients for parents of sons than for parents of daughters. Parents’ gender-typing of stereotypically feminine and stereotypically masculine toys were highly correlated in parents of sons. Therefore, we carried out all further analyses with the newly formed scales depicting parents’ desirability judgments about same- and cross-gender-typed toys and their overall gender-typing of toys.

**Table 1:** Correlations between original study variables for parents thinking of sons and daughters

Variables	Correlations					
	1	2	3	4	5	6
1. Desirability of stereotypical girls’ toys	–	−.25**	.59**	−.51**	−.35**	−.18*
2. Desirability of stereotypical boys’ toys	−.15	–	.09	.05	.11	−.05
3. Desirability of gender-neutral toys	.29**	.28**	–	−.34**	−.25**	−.18*
4. Gender-typing of stereotypical girls’ toys	.05	−.30**	−.13	–	.74**	.43**
5. Gender-typing of stereotypical boys’ toys	.12	−.39**	−.10	.61**	–	.49**
6. NGRO	−.13	−.17*	−.20**	.29**	.34**	–

Correlation coefficients for parents thinking of daughters are reported below the diagonal; parents thinking of sons, above the diagonal. NGRO = Normative Gender Role Attitudes. Desirability judgments range from 1 to 7, with higher values indicating higher desirability of a given type of toy; Gender-typing scores range from 1 to 100, with higher values indicating stronger gender-typing of toys; NGRO scores range from 1 to 7, with higher values indicating more traditional gender role attitudes

*p < .05. ^**^p < .01

### Parents’ Toy Desirability Judgments

We expected parents to judge same-gender-typed toys as more desirable for their children than gender-neutral or cross-gender typed toys (Hypothesis 1). A repeated measures ANOVA showed significant differences in parents’ judgments of the desirability of the different types of toys, *F*(2, 322) = 340.83, *p* < .001, ηp^2^ = .513. Planned contrasts revealed that parents rated same-gender-typed toys, *F*(1, 323) = 313.33, *p* < .001, ηp^2^ = .492, and gender-neutral toys, *F*(1, 323) = 749.10, *p* < .001, ηp^2^ = .699, as more desirable for their children than cross-gender-typed toys, but they showed no differences in parents’ desirability judgments for same-gender-typed and gender neutral toys, *F*(1, 323) = 3.67, *p* < .056, ηp^2^ = .011. These results are partly in line with our hypothesis. Table 2 shows the mean ratings for the different types of toys including their standard deviations and confidence intervals.

**Table 2:** Descriptive statistics and correlations among toy desirability judgments, gender-typing of toys, and gender role attitudes

Variables	*M* (*SD*)	95% CI	Correlations				
			1	2	3	4	5
1. Desirability of same-gender-typed toys	5.15 (1.16)	[5.02, 5.28]	–	−.22^**^	.20^**^	.09	−.07
2. Desirability of cross-gender-typed toys	3.14 (1.45)	[2.98, 3.30]		–	.41^**^	−.42^**^	−.18^**^
3. Desirability of gender-neutral toys	5.30 (1.13)	[5.18, 5.42]			–	−.22^**^	−.18^**^
4. Gender-typing of toys	71.38 (13.72)	[69.88, 72.88]				–	.41^**^
5. NGRO	2.98 (.96)	[2.88, 3.09]					–

NGRO = Normative Gender Role Attitudes. Desirability judgments range from 1 to 7, with higher values indicating higher desirability of a given type of toy; Gender-typing scores range from 1 to 100, with higher values indicating stronger gender-typing of toys; NGRO scores range from 1 to 7, with higher values indicating more traditional gender role attitudes. Toy desirability judgments and gender-typing of toys are converted scores

**p < .01

We examined simple correlations between parents’ toy desirability judgments, gender-typing of toys, and NGRO (see Table 2). We expected more traditional gender role attitudes in parents to be associated with higher desirability judgments of same-gender-typed toys and lower desirability judgments of cross-gender-typed toys for their children (Hypothesis 2). Contrary to our expectations, we found no significant correlation between parents’ NGRO and their desirability judgments about same-gender-typed toys. This indicates that parents’ judgments about the desirability of same-gender-typed toys for their children are not associated with the traditionalism of their gender role attitudes. In contrast, parents’ NGRO was negatively correlated with their desirability judgments about cross-gender-typed toys. This is in line with our expectations and suggests that traditional gender role attitudes are associated with the rejection of cross-gender-typed toys.

As expected in Hypothesis 3, parents’ NGRO was positively correlated with their gender-typing of toys, showing that parents with a more traditional view of gender roles see stereotypically feminine toys as more appropriate for girls and stereotypically masculine toys as more appropriate for boys than do parents with a more egalitarian view of gender roles. It also affirms that NGRO, which is a measure designed to assess individuals’ attitudes about men’s and women’s suitability for different roles, occupations, and domestic and family responsibilities, extends to attitudes about children’s toys.

To test Hypotheses 4 and 5, we conducted a multivariate analysis of covariance (MANCOVA) that examined age and educational level as covariates, gender of parent and gender of child as independent variables, and parents’ desirability judgments of different types of toys (same-gender-typed, cross-gender-typed, and gender-neutral), gender-typing of toys, and NGRO as dependent variables. The MANCOVA yielded significant multivariate effects for the covariates of parents’ age, *F*(5, 314) = 4.45, *p* = .001, ηp^2^ = .066, and education, *F*(5, 314) = 15.31, *p* < .001, ηp^2^ = .196. It also showed significant main effects for gender of parent, *F*(5, 314) = 3.11, *p* = .009, ηp^2^ = .047, and gender of child, *F*(5, 314) = 5.04, *p* < .001, ηp^2^ = .074. The interaction between gender of parent and gender of child was not significant, *F*(5, 314) = .99, *p* = .42. We followed up the significant multivariate effects with univariate analyses of variance (ANOVAs), conducting a Holm-Bonferroni sequential correction in order to control for the family-wise error rate (Holm 1979). Table 3 shows the mean scores of parents’ toy desirability judgments for their children, gender-typing of toys, and gender role attitudes by gender of parent and gender of child, and it lays out cell sizes.

**Table 3:** Toy desirability judgments, gender-typing of toys, and gender role attitudes by parent’s and child's gender

Variables	Gender of parent	Gender of child	*M* (*SD*)	*n*
Desirability of same-gender-typed toys	Female	Male	5.01 (1.25)	120
		Female	5.34 (1.09)	136
		Total	5.18 (1.18)	256
	Male	Male	4.85 (1.11)	35
		Female	5.19 (1.07)	33
		Total	5.01 (1.09)	68
	Total	Male	4.97 (1.22)	155
		Female	5.31 (1.08)	169
		Total	5.15 (1.16)	324
Desirability of cross-gender-typed toys	Female	Male	3.36 (1.47)	120
		Female	2.93 (1.41)	136
		Total	3.13 (1.45)	256
	Male	Male	3.30 (1.54)	35
		Female	3.01 (1.40)	33
		Total	3.16 (1.47)	68
	Total	Male	3.35 (1.48)	155
		Female	2.95 (1.40)	169
		Total	3.14 (1.45)	324
Desirability of gender-neutral toys	Female	Male	5.25 (1.17)	120
		Female	5.48 (1.09)	136
		Total	5.37 (1.13)	256
	Male	Male	4.81 (1.29)	35
		Female	5.28 (.82)	33
		Total	5.04 (1.10)	68
	Total	Male	5.15 (1.21)	155
		Female	5.44 (1.04)	169
		Total	5.30 (1.13)	324
Gender-typing of toys	Female	Male	71.46 (13.56)	120
		Female	70.82 (13.78)	136
		Total	71.12 (13.65)	256
	Male	Male	71.88 (14.31)	35
		Female	72.92 (13.98)	33
		Total	72.39 (14.06)	68
	Total	Male	71.55 (13.69)	155
		Female	71.23 (13.80)	169
		Total	71.38 (13.72)	324
NGRO	Female	Male	2.83 (.90)	120
		Female	2.99 (.95)	136
		Total	2.91 (.93)	256
	Male	Male	3.06 (.97)	35
		Female	3.39 (1.14)	33
		Total	3.22 (1.06)	68
	Total	Male	2.88 (.92)	155
		Female	3.07 (1.00)	169
		Total	2.98 (.96)	324

NGRO = Normative Gender Role Attitudes. Desirability judgments range from 1 to 7, with higher values indicating higher desirability of a given type of toy; Gender-typing scores range from 1 to 100, with higher values indicating stronger gender-typing of toys; NGRO scores range from 1 to 7, with higher values indicating more traditional gender role attitudes. The smallest cell size was 33 for fathers thinking of daughters. Toy desirability judgments and gender-typing of toys are converted scores

The multivariate effect for age was accounted for by a univariate effect for NGRO, *F*(1, 318) = 15.37, *p* < .001, ηp^*2*^ = .046. Two univariate effects underscored the multivariate effect for educational level: gender-typing of toys, *F*(1, 318) = 14.69, *p* < .001*, ηp*^*2*^ *=* .044; and NGRO, *F*(1, 318) = 63.35, *p* < .001*, ηp*^*2*^ *=* .166. The significant univariate effects for parents’ gender were for NGRO, *F*(1, 318) = 10.84, *p* = .012, ηp^2^ = .033. Finally, for the main effect of gender of child, none of the univariate effects was significant after the Holm-Bonferroni sequential correction. All univariate test statistics, including the unadjusted and adjusted *p*-values can be found in an online supplement (see Table 3s).

Turning to how these findings relate to our hypotheses, the first part of Hypothesis 4 predicted that younger parents would exhibit more egalitarian toy desirability judgments, less gender-typing of toys, and higher levels of egalitarian attitudes than would older parents. Our hypothesis was not supported for toy desirability judgments and gender-typing of toys because we did not find any differences between parents of different age here. For gender role attitudes, we found a significant effect but not in the expected direction. Younger parents reported more traditional gender role attitudes than did older parents. The second part of Hypothesis 4 predicted that parents with higher educational levels would report more egalitarian toy desirability judgments, less gender-typing of toys, and higher levels of egalitarian attitudes than parents with lower educational attainment would. This hypothesis was supported for gender-typing of toys and for gender-role attitudes but not for toy desirability judgments. Moreover, we hypothesized that mothers would exhibit more egalitarian toy desirability judgments, less gender-typing of toys, and higher levels of egalitarian attitudes than fathers would. Our findings supported this hypothesis only for gender-role attitudes but not for toy desirability judgments or gender-typing of toys.

In Hypothesis 5, we expected parents to judge cross-gender-typed toys as less desirable for sons than for daughters. Contrary to our hypothesis, there was no significant difference in desirability judgments of cross-gender-typed toys between parents thinking of sons and parents thinking of daughters while answering the questionnaire.

## Discussion

The present study aimed at gaining greater insight into parents’ role in encouraging children’s gender-stereotyped toy play by investigating different aspects of parents’ gender-related attitudes that are assumed to relate to the three major modes of influence on children’s gender socialization in social-cognitive theory (Bussey and Bandura 1999). For this purpose, we used three measures, two of which utilized toys as stimulus material. In a pilot study, we also identified a concise set of toys currently seen as stereotypically feminine, gender-neutral, and stereotypically masculine. Except for plasticine, which is a very common toy in German-speaking countries but has not appeared in previous studies, the gender-typing of toys in our study was closely aligned with previous findings on the gender-typing of toys (Blakemore and Centers 2005; Campenni 1999).

In the main study, participants were first asked to indicate how desirable they found each toy for their own child in order to assess their judgments about the desirability of different types of toys for their children. At that time, the participants did not know about the objectives of the study and had not yet answered any gender-related questions. Therefore, this measure allowed us to compare the extent to which parents of daughters and parents of sons endorsed different types of toys for their child without triggering the social desirability of egalitarian attitudes. We assumed parents’ toy desirability ratings to be an indicator for their reactions to their children playing with the respective toy type and their toy choices for their children. Parents who judge a toy as undesirable for their child would probably not react in a positive way to seeing their child play with it and would probably not purchase it for their child. Second, parents were asked to rate the appropriateness of the 15 toys for girls and boys (i.e. to indicate their gender-typing of these toys). Rating the appropriateness of toys for boys and girls required parents to explicitly apply their gender-stereotyped attitudes (i.e. their beliefs about the typical interests, competences and roles of boys and girls). We assumed parents’ gender-typing of toys to be an indicator for the gender-related attitudes about toys they directly communicate to their children. Third, we assessed the participants’ gender role attitudes via self-reports (Athenstaedt 2000). Because egalitarian parents were found to divide domestic labor more fairly than traditional parents (Crouter et al. 1995; Greenstein 1996; Updegraff et al. 1996), we assumed parents’ gender role attitudes to be an indicator for the gender roles they model.

We conducted preliminary analyses to find out whether the patterns of associations between the measures differed between parents of daughters and parents of sons because Blakemore and Hill (2008) found a considerably weaker and somewhat counterintuitive pattern in parents of daughters. In our study, we did not find different patterns of associations for parents of daughters and parents of sons. Therefore, we conducted all further analyses with scales depicting the desirability of same-gender-typed and cross-gender-typed toys to ensure greater clarity and easier interpretation.

We examined parents’ judgments about the desirability of the different types of toys for their children because we assumed these judgments to be an indicator for parents’ toy choices and reactions to their children’s toy choices and play. Although parents judged same-gender-typed and gender-neutral toys as equally desirable, they rated cross-gender-typed toys as considerably less desirable for their children. This result is not in line with our hypothesis that parents would see same-gender-typed toys as more desirable than gender-neutral toys and cross-gender-typed toys (Fisher-Thompson 1993; Kim 2002). However, our finding corresponds to a study by Dinella et al. (2017), in which children were more interested in gender-typed and gender-neutral toys than in cross-gender-typed toys. Parental rejection of cross-gender-typed toys may thus be a more important factor in children’s construction of personal standards regarding toys than parents’ endorsement of gender-typed toys. Parents who do not want their children to play with cross-gender-typed toys limit their children’s action repertoire in accordance with gender stereotypes. Ancillary correlation results (see Table 2) showed that parents who endorsed same-gender-typed toys for their children rejected cross-gender-typed toys and vice versa, whereas gender-neutral toys were endorsed by parents regardless of their desirability judgments of same-gender-typed and cross-gender-typed toys. This pattern corresponds to findings that boys and girls differ in their preferences for play with stereotypically masculine and stereotypically feminine toys, but not with gender-neutral toys (Li and Wong 2016).

Next, we investigated associations between different aspects of parents’ gender-related attitudes to contribute to a better understanding of their interplay in the parental transmission of gender stereotypes. Parents’ gender role attitudes were not associated with their desirability judgments of gender-typed toys for their children but rather with their desirability judgments of cross-gender-typed toys. Parents with egalitarian gender role attitudes found cross-gender-typed toys more desirable for their children than did parents with traditional gender role attitudes, which is in line with our hypothesis. This finding indicates that egalitarian parents permit a greater range of interests and behaviors in their children than traditional parents do by creating a less stereotypical environment for them. Interestingly, they seem to do so not by discouraging children’s interests in same-gender-typed toys but by accepting children’s interest in cross-gender-typed toys to a greater extent. This relates to the importance of the personal self-regulative standards children develop from information conveyed via the three principal modes of influence for gender development and differentiation (Bussey and Bandura 1999).

Children whose parents permit more diverse play options may not develop equally strict gender-stereotyped standards as children whose parents heavily sanction interest in cross-gender-typed toys. This may be one reason for children with egalitarian parents learning gender stereotypes later than children with traditional parents (Fagot and Leinbach 1995). Our findings are only partly consistent with those of Blakemore and Hill (2008), who found parents’ gender role attitudes to be related to parents’ endorsement of both same- and cross-gender-typed toys, albeit with different relationship patterns for parents of daughters and parents of sons. This discrepancy could be explained by the different measures used in the two studies. Blakemore and Hill used measures of sexism, included only one gender-neutral toy, and examined parents’ endorsement of gender-typed toys and activities simultaneously whereas we used a measure of gender role attitudes, included five gender-neutral toys, and examined parents’ endorsement of gender-typed toys but not of gender-typed activities.

Parents’ gender role attitudes and their gender-typing of toys were positively associated, such that parents with more traditional gender role attitudes reported stronger gender-typing of toys than did parents with more egalitarian gender role attitudes. This indicates that parents hold a constellation of consistent attitudes about gender regarding roles and features of toys. To our knowledge, no previous research has investigated the relationship between parents’ gender role attitudes and their gender-typing of toys. Nevertheless, this result is in line with our hypothesis. It is plausible that parents who endorse traditional gender roles for adults also see toys that allow traditional gender roles to be practiced as more appropriate for children. If parents’ gender-typing of toys is an indicator for their direct communication with their children about toys and gender, this result could be interpreted such that traditional parents not only model more traditional gender roles but also communicate stronger gender-typing of toys to their children than egalitarian parents do.

We examined individual differences in parents’ toy desirability judgments, gender-typing of toys, and gender role attitudes based on parents’ gender, age and educational levels as well as child’s gender. Regarding parents’ gender, fathers were found to have more traditional views on gender roles than mothers do, which is in line with previous studies (Apparala et al. 2003; Athenstaedt 2000; Donnelly et al. 2015; Harris and Firestone 1998; Kulik 2002). However, we did not find differences between mothers and fathers in the gender-typing of toys or toy desirability judgments. It appears that the categories of toys identified as appropriate for girls and boys are generally agreed upon as standards and that mothers and fathers do not differ in their judgments about the desirability of different types of toys for their children. This relates to findings that both mothers and fathers are more likely to buy gender-typed than non-gender-typed toys (Fisher-Thompson 1993) and that mothers and fathers equally encourage gender-typed activities in their children (Lytton and Romney 1991).

Parents’ age was significantly related to their gender role attitudes but not in the expexted way. Older parents had more egalitarian views on gender roles than younger parents had, although higher age has often been associated with more traditional gender role attitudes in previous studies (Athenstaedt 2000; Calvo-Salguero et al. 2008; Harris and Firestone 1998; Kulik 2002). The present study’s contrasting result may be explained by characteristics of the parent population. Presumably, individuals with more traditional gender role attitudes may decide to have children earlier than those with more egalitarian gender role attitudes. Women with egalitarian attitudes often pursue their career before having children, which may result in older parents having more egalitarian gender role attitudes than younger parents. Parents’ gender-typing of toys and their toy desirability judgments did not differ depending on their age.

Regarding educational attainment, analyses revealed that parents with higher educational levels reported more egalitarian gender role attitudes, which is in line with previous research (Apparala et al. 2003; Athenstaedt 2000; Donnelly et al. 2015; Harris and Firestone 1998; Kulik 2002). This finding supports the conclusion that higher age might be related to more egalitarian attitudes because older parents pursue their career before having children. Higher educational level was also associated with less gender-typing of toys, which is plausible given the association between gender role attitudes and gender-typing of toys. Surprisingly, no differences in toy desirability judgments were found between parents with different educational backgrounds. This indicates that parents’ judgments about the desirability of different toys for their children are less influenced by their formal education than by their gender role attitudes and tendency to see toys as gender-typed. The fact that gender stereotypes are socially unacceptable in highly-educated milieus may also contribute to discrepancies in different indicators for gender-related attitudes varying in face validity (Freeman 2007; Kamo 2000; White and White 2006).

Parents of sons and parents of daughters did not differ in their toy desirability judgments. This is contrary to our hypothesis derived from findings that boys engaging in cross-gender-typed play tend to be criticized more heavily than girls (Cahill and Adams 1997; Martin 1990, 1995). However, these previous findings were obtained over 20 years ago and might therefore be outdated.

### Limitations and Future Research Directions

The most important limitation of the present study is that it does not include child-level data. Future research should consider children’s toy preferences, gender-typing of toys, and gender role attitudes to gain more insight into parents’ role in children’s gender socialization. This would allow for an investigation of which aspects of parents’ gender-related attitudes and behaviors affect children’s gender-related cognitions and behaviors, as well as to what extent. Moreover, we did not assess parental behavior (i.e. their actual toy selection, reactions to children’s toy play and choices, direct tuition and modelling; Bussey and Bandura 1999), but only their judgments about the desirability of different types of toys, their gender-typing of toys, and their gender-role attitudes. Although it seems plausible that parents make the toys they find desirable for their children available to them, react more positively when their children play with toys they find more desirable, communicate their gender-typing of toys to their children, and model behavior that corresponds with their gender role attitudes, further research should investigate whether this is actually the case.

Another limitation of the present study is that it is not clear exactly what underlies parents’ choice of certain toys as “desirable” for their child. There are different possible explanations for the finding that parents reported low desirability of cross-gender-typed toys for their child. Parents might think that their reports reflect their children’s toy preferences (Etaugh and Liss 1992; Francis 2010), which would indicate that this question is not a measure of parents’ attitudes in the narrower sense. They may also be afraid that their children would be laughed at or excluded when playing with cross-gender-typed toys (Ruble et al. 2007) or they have (conscious or unconscious) misgivings when they think about their own child playing with cross-gender-typed toys. Future research should therefore examine the motives behind parents’ toy desirability judgments for their children using qualitative methods like interviews or focus groups. A better understanding of parents’ motives for not providing their children with cross-gender-typed toys is crucial for designing interventions dealing with this issue.

### Practice Implications

The present study shows that parents judge gender-typed and gender-neutral toys as more desirable for their children than cross-gender-typed toys regardless of the parents’ gender, age, and educational level. This indicates that even boys and girls from highly educated families are still confronted with differential expectations regarding roles, interests, and skills that may lead to unused chances and limited action repertoires for both girls and boys. Implementing interventions for parents is challenging because parents are hard to reach. Preschools, however, are promising starting points for enacting change in parents and preschool teachers. Previous research found that preschool teachers project gender prejudices about play onto their students (Lynch 2015) and that children in preschools with a focus on gender-neutral education were found to show less gender stereotyping than children in typical preschools (Shutts et al. 2017). Thus, training programs on gender-sensitive pedagogy for preschool teachers that include parental outreach may be an important step in promoting gender fairness in education.

In Austria, the Federal Ministry of Families and Youth facilitated the development of such a training program (Kollmayer et al. 2016). In four modules, preschool teachers learn about children’s gender development and differentiation, mechanisms and materials that perpetuate gender stereotypes in preschool settings, and how to create a less stereotypical environment that enables a greater range of interests and behaviors in children. Between the modules, preschool teachers work on reflection exercises that help them integrate the content of the training into their day-to-day work, reflect on opportunities and obstacles, and practice their role as multipliers within their own preschool. One module of the training program focuses on gender-sensitive parental outreach. In this module, the teachers design a parent-teacher conference dealing with gender stereotypes in preschools. In role plays, the teachers also practice how to address gender stereotypes in outreach to parents without triggering resistance. Such programs may help parents reflect on their toy desirability judgments and on the possible effects of their toy choices and reactions to their children’s toy play.

### Conclusion

Although parental toy selection and responses to toy play are considered important factors in children’s gender socialization, little is known about the factors that underlie parents’ judgments about the desirability of different types of toys for their children. Therefore, the present study investigated how parents’ judgements about the desirability of same-gender-typed, cross-gender-typed, and gender-neutral toys relate to their gender-typing of toys, gender role attitudes, and demographics.

The most important finding of the present study was that even though gender role attitudes are becoming more and more egalitarian, parents still find same-gender-typed and gender-neutral toys more desirable for their children than cross-gender-typed toys. This pattern is consistent for mothers and fathers of sons and daughters irrespective of parents’ age, gender, and educational levels. Parents who do not make cross-gender-typed toys available for their children restrict the roles their children can practice in their play activities and thereby promote the development of gender-stereotypical attributes in their children. Interestingly, the traditionalism of parents’ gender role attitudes was not associated with their desirability judgments of same-gender-typed toys for their children but with their desirability judgments of cross gender-typed toys and their overall tendency to see toys as gender-typed. Parents with egalitarian gender role attitudes perceived cross-gender-typed toys as more desirable for their children and reported a lower level of gender-typing of toys then parents with traditional gender role attitudes did. Thus, egalitarian parents seem to permit a greater range of interests and behaviors in their children than traditional parents do. Summing up, the present study highlights the importance of investigating different aspects of parents’ gender-related attitudes simultaneously. Discovering discrepancies between different aspects of parents’ gender-related attitudes can contribute to a better understanding of parents’ complex influence on children’s gender socialization and differentiation.

## Electronic supplementary material

ESM 1 (DOCX 38 kb)
