# Supplementary material for: Parents’ Judgments about the Desirability of Toys for Their Children: Associations with Gender Role Attitudes, Gender-typing of Toys, and Demographics
Source: Sex Roles. 2018 Jan 13;79(5):329–41. doi: 10.1007/s11199-017-0882-4 (PMC6096664; doi:10.1007/s11199-017-0882-4)
Supplement: Supplementary file 1 — (DOCX 38 kb) [file 11199_2017_882_MOESM1_ESM.docx]

Online supplement for Kollmayer, M., Schultes, M-T., Schober, B., Hodosi, T., and Spiel, C. (2017). Parents’ judgments about the desirability of toys for their children: Associations with gender role attitudes, gender-typing of toys, and demographics. Marlene Kollmayer, University of Vienna. Email: [marlene.kollmayer@univie.ac.at](mailto:marlene.kollmayer@univie.ac.at)

Table 1s

*Gender-typing of toys by 29 Community Participants in the Pilot Study*

| Toy | *M* | *SD* |
| --- | --- | --- |
| Barbie doll^a^ | 7.96 | 8.56 |
| Wooden beads^a^ | 17.28 | 15.80 |
| Doll clothes^a^ | 19.79 | 17.12 |
| Doll house^a^ | 22.90 | 18.18 |
| Doll buggy^a^ | 23.34 | 17.93 |
| Sewing machine | 28.69 | 21.03 |
| Baby bottle | 29.38 | 18.96 |
| Doll | 29.79 | 17.84 |
| Tea set | 31.28 | 17.82 |
| Frame loom | 33.10 | 18.71 |
| Flat iron | 36.48 | 13.23 |
| Stove | 37.72 | 16.95 |
| Toy kitchen | 37.86 | 16.97 |
| Vacuum cleaner | 38.34 | 17.61 |
| Hand puppet | 41.69 | 14.52 |
| Teddy bear | 47.90 | 8.49 |
| Coloring book | 48.48 | 4.20 |
| Jigsaw puzzle | 49.28 | 4.33 |
| Xylophone^b^ | 49.69 | 2.41 |
| Doctor’s kit^b^ | 49.83 | 7.38 |
| Alphabet puzzle^b^ | 49.83 | 7.60 |
| Magnetic drawing board^b^ | 50.00 | 0.00 |
| Plasticine^b^ (modeling clay) | 50.97 | 2.88 |
| Clock | 51.45 | 6.89 |
|  | | |
|  |  |  |
|  |  |  |
| Toy | *M* | *SD* |
|  |  |  |
| Trampoline | 51.86 | 6.55 |
| Animal figures | 52.14 | 8.32 |
| Lego | 54.07 | 9.43 |
| Scooter | 54.45 | 7.98 |
| Locomotive | 62.55 | 17.09 |
| Gas station | 65.59 | 13.76 |
| Garage | 68.72 | 17.26 |
| Remote controlled car | 69.93 | 16.56 |
| Race course | 71.00 | 18.21 |
| Toolbox | 71.10 | 17.51 |
| Police uniform | 71.41 | 20.96 |
| Cowboy | 71.52 | 17.12 |
| Spiderman | 72.97 | 18.03 |
| Matchbox car^c^ | 73.14 | 16.38 |
| Boxing gloves^c^ | 74.14 | 19.35 |
| Helicopter^c^ | 76.17 | 17.25 |
| Truck^c^ | 77.55 | 15.54 |
| Transformer^c^ | 78.62 | 18.79 |

*Note*. Ratings from 29 raters ranged from 0 = *for girls* to 100 = *for boys*.

^a^Toys used as “for girls” in the present study. ^b^Neutral toys. ^c^”for boys.”

Table 2s

*Gender-typing of Toys by 324 Parents in the Main Study*

|  |  | *M* | *SD* |
| --- | --- | --- | --- |
| Stereotypical girls’ toys | Barbie doll | 15.84 | 18.98 |
|  | Doll buggy | 25.05 | 20.65 |
|  | Doll clothes | 30.04 | 22.08 |
|  | Doll house | 35.27 | 19.80 |
|  | Wooden beads | 38.83 | 17.51 |
| Gender-neutral toys | Doctor’s kit | 49.10 | 6.87 |
|  | Plasticine (molding clay) | 49.16 | 5.54 |
|  | Xylophone | 49.22 | 5.08 |
|  | Alphabet puzzle | 49.91 | 0.89 |
|  | Magnetic drawing board | 50.06 | 4.97 |
| Stereotypical boys’ toys | Matchbox car | 66.75 | 20.40 |
|  | Truck | 68.48 | 20.85 |
|  | Helicopter | 69.59 | 21.48 |
|  | Boxing gloves | 76.35 | 21.61 |
|  | Transformer | 78.08 | 20.27 |

*Note*. Values range from 0 = *for girls* to 100 = *for boys*.

Table 3s

*Effects of Parents’ Age, Educational Level, and Gender, and of Child’s Gender on Parents’ Toy Desirability Judgments for their Children, Gender-Typing of Toys and Gender Role Attitudes*

| Independent Variable | Dependent Variable | *F* | *p* | *p*_adj_ | ηp^2^ |
| --- | --- | --- | --- | --- | --- |
| Age | Desirability of same-gender-typed toys | 1.15 | .284 | .932 | .004 |
|  | Desirability of cross-gender-typed toys | 0.86 | .354 | .932 | .003 |
|  | Desirability of gender-neutral toys | 1.41 | .236 | .932 | .004 |
|  | Gender-typing of toys | 0.31 | .581 | .999 | .001 |
|  | NGRO | 15.37 | .000 | .000 | .046 |
| Educational level | Desirability of same-gender-typed toys | 6.21 | .013 | .143 | .019 |
|  | Desirability of cross-gender-typed toys | 2.89 | .090 | .450 | .009 |
|  | Desirability of gender-neutral toys | 0.65 | .421 | .999 | .002 |
|  | Gender-typing of toys | 14.69 | .000 | .000 | .044 |
|  | NGRO | 63.35 | .000 | .000 | .166 |
| Gender | Desirability of same-gender-typed toys | 1.43 | .233 | .932 | .004 |
|  | Desirability of cross-gender-typed toys | 0.01 | .914 | .999 | .000 |
|  | Desirability of gender-neutral toys | 4.99 | .026 | .230 | .015 |
|  | Gender-typing of toys | 0.28 | .595 | .999 | .001 |
|  | NGRO | 10.84 | .001 | .012 | .033 |
| Gender of child | Desirability of same-gender-typed toys | 4.74 | .030 | .230 | .015 |
|  | Desirability of cross-gender-typed toys | 3.49 | .063 | .378 | .011 |
|  | Desirability of gender-neutral toys | 5.17 | .024 | .230 | .016 |
|  | Gender-typing of toys | 0.02 | .877 | .999 | .000 |
|  | NGRO | 5.24 | .023 | .230 | .016 |

*Note*. NGRO = Normative Gender Role Attitudes. Column *p* shows the unadjusted *p*-values, column *p*_adj_ shows the adjusted *p*-values after Holm-Bonferroni sequential correction.
